# Markers of Biological Stress and Mucosal Immunity during a Week Leading to Competition in Adolescent Swimmers

**DOI:** 10.1155/2014/234565

**Published:** 2014-06-12

**Authors:** E. Papadopoulos, C. Muir, C. Russell, B. W. Timmons, B. Falk, P. Klentrou

**Affiliations:** ^1^Department of Kinesiology, Faculty of Applied Health Sciences, Brock University, St Catharines, ON, Canada L2S 3A1; ^2^Department of Psychology, Centre for Neuroscience, Brock University, St Catharines, ON, Canada L2S 3A1; ^3^Department of Pediatrics, McMaster University, Hamilton, ON, Canada L8S 4L8

## Abstract

In this study we examined changes in the salivary concentrations of immunoglobulin A (sIgA), cortisol (sC), testosterone (sT), and testosterone-to-cortisol ratio (T/C) in 21 competitive swimmers, 11–15 years old, during a week leading to competition as compared to a control (noncompetition) week. No day-to-day changes or significant differences between weeks were observed for sIgA (47.9 ± 4.4 versus 54.9 ± 5.2 *μ*g/mL for control versus competition week, resp.), sC (2.7 ± 0.2 versus 2.5 ± 0.2 ng/mL for control versus competition week, resp.), and T/C ratio (83.4 ± 7.0 versus 77.9 ± 7.7 for control versus competition week, resp.). In contrast, sT was significantly lower during the week of competition (154.5 ± 11.3 pg/mL) as compared to the control week (181.3 ± 11.5 pg/mL) suggesting that the swimmers were in a catabolic state, although this did not have a negative effect on their performance. In conclusion, salivary cortisol did not change between the two weeks, and thus competition stress was relatively low, and mucosal immunity was unaffected in these young athletes prior to competition.

## 1. Introduction

Young, competitive swimmers can be subject to intense training. This may have a potential effect on their mucosal immunity since there is some evidence in the literature that long periods of training can lead to immunosuppression in adult swimmers [1–3]. In particular, salivary Immunoglobulin A (sIgA), the most abundant marker of mucosal immunity, has been shown to decrease in response to prolonged training in adult swimmers [4]. However, another study found no significant changes in sIgA after 15 weeks of training in elite swimmers [5] while there are no such studies in adolescent swimmers.

Competition events have been shown to increase stress, reflected by higher cortisol levels [6, 7]. According to Filaire et al. [6], salivary cortisol increased during a major competition in judo athletes, with a similar response reported for volleyball and basketball players prior to a significant match [7, 8].  All these studies measured the levels of salivary cortisol and other stress hormones without taking into account the variability of these hormones. On the other hand, people with high hormonal variability may be more adaptive and able to cope with situations such as stress; thus, the day-to-day variability of stress hormones (as measured by the % coefficient of variation) may be a better indication of adaptability than a single measurement [9, 10].

The interaction between stress and immune markers due to competition is not clear, especially in child athletes. He et al. [8] reported an inverse relationship between sIgA and cortisol levels in adult basketball players during competition periods, but there are no relevant studies that examine the potential chronic effect of stress on immunity markers in the days leading to a competition in young athletes. As the number of children and adolescents that engage in high performance sports at a steadily younger age has increased, it is important to investigate any potential health implications of youth sport participation. Studies on the cumulative effect of training and competition on young athletes' health and performance are, therefore, timely. The purpose of this study was to determine whether the combination of training and competition stress has an effect on the salivary concentration and variability of stress hormones in young swimmers during a week leading to competition, as compared to a control (noncompetitive) week, and if this is associated with changes in their mucosal immunity. It was hypothesized that there would be an elevated stress response with sC levels and variability gradually increasing and the T/C ratio decreasing as competition approached and that these changes will be associated with a decrease in sIgA prior to competition.

## 2. Materials and Methods

### 2.1. Participants

The present study and all related procedures received ethical clearance from the Brock University Research Ethics Board. Thirty competitive swimmers, 11–15 years old, who did not receive a flu shot in the preceding 12 months, were recruited from swimming clubs across Southwest Ontario and invited to participate in the study.

### 2.2. Study Design

The study was conducted between the end of November and mid-January, to avoid seasonal variations in the outcome measures. Researchers met with the young athletes and their parents one week prior to data collection. During this meeting, the participants were provided with a detailed description of the study. A consent form was then completed and signed by the parents of the athletes who agreed to participate in the study. Subsequently, anthropometric and skinfold measurements were taken followed by the completion of a questionnaire package. The questionnaire package included information concerning medical history and sexual maturity. A health log was then handed out to each participant to record any upper respiratory tract infection (URTI) symptoms during each of the two weeks of the study. All questionnaires were completed by the swimmers.

Swimmers were then asked to self-collect one resting saliva sample every morning for one control, noncompetition week, and every morning for a week leading to and including an important competition. The same procedure was followed both weeks with collection starting on Sunday and continuing for 7 days until Saturday. This means that the competition week included two days of competition (Friday and Saturday). To ensure that there was no cross-contamination in the hormonal levels from one week to the next, the two weeks were at least one week apart. The competition was similarly important for all swimmers in that all swimmers had to achieve specific qualifying standards in order to advance to the next competitive level.

### 2.3. Measurements

*Anthropometry*. Body mass (kg) and height (cm) were assessed using a weight scale and a stadiometer, respectively. Relative body fat (%BF) was estimated using skinfold thickness (mm) assessed at two sites, the arm and upper back (triceps and subscapular), as previously described [11]. The same investigator completed all anthropometric measures for all participants.

*Sexual Maturation*. Sexual maturity was self-assessed according to pubic hair development (TPH), as defined by Tanner [12, 13]. Tanner staging is a well-accepted method to assess sexual maturity in pediatric samples [14, 15]. Female participants were also asked to indicate whether or not they had reached menarche, and if so, at what age.

*Competition Anxiety*. Competition anxiety was self-reported using the Sport Anxiety Scale 2 (SAS-2), which is a multidimensional measure of cognitive and somatic trait anxiety for children in sport performance settings [16]. In order to ensure consistency in the responses, subjects were asked to fill the SAS-2 twice, once during the control week and on the day (day 5) prior to competition.

*URTI Incidence*. The frequency of URTI during each week was determined using a daily health log as previously described [17, 18]. URTIs were recorded in order to (a) identify those swimmers who may have high levels of sIgA due to a potential URTI, and (b) examine if stress correlates with URTIs independently of sIgA. The log quantifies the frequency and duration (number of days) of URTI symptoms. Participants were asked to record any cold and flu symptoms each day of the week using a set of codes provided with the log. This method was chosen to eliminate participant bias when recording from memory. The parents and/or participants were asked to return the health log once completed. The total number of days per week with URTI symptoms was then tallied for each participant, with days being counted only if two or more consecutive days of cold or flu symptoms were reported [17, 18].

*Training and Performance Data*. During the control week, the swimmers trained 14–19 hours/week (12–16 hours of swimming and 3-4 hours of land/weight training). During the experimental/competition week, the swimmers trained 8.5–9.5 hours/week (6.5–7.5 hours of swimming and 1-2 hours of land/weight training). Thus, training volume (defined as hours per week) was reduced during the competition week by an average of 25% amongst the different groups/teams. Performance data were obtained from the official competition results, and the difference between seeding and final times was used to quantify each swimmer's performance outcome.

*Saliva Collection and Analysis*. To ensure consistency and to account for diurnal fluctuation in hormones, all saliva samples were obtained in the morning upon awakening. Specifically, all swimmers were provided with salivette swabs in order to self-collect one milliliter of unstimulated whole mixed saliva each morning upon awakening, before breakfast and before brushing their teeth. They were provided with written instructions of how to collect the saliva sample. The swab was placed in the mouth for one minute and then transferred directly into the plastic tubes and stored in −20°C until practice time, at which time tubes were submitted to the research team. Once all samples of each week were collected, the tubes were centrifuged at 3000 ×g for 10 minutes where impurities were filtered out and the resulted saliva sample was aliquoted into two separate 1.5 mL eppendorf tubes and stored at −80°C until analysis.

Mucosal sIgA was assayed in duplicate by commercially available ELISA kits (Salimetrics, LLC, Pennsylvania, USA). Due to recent evidence that concentrations of sIgA, as measured by ELISA, may be affected by the use of salivettes [19], a normal set of standards was compared with a second set of standards (10 *μ*L of standard in 4 mL of 1X sIgA diluent) that was run through a set of six salivettes and centrifuged for 10 minutes at 3500 rpm to separate the standards from the swabs. The two sets of standards were then compared and found to be no different. Cortisol and testosterone were assayed using an in-house assay in the Department of Psychology at Brock University using methods described elsewhere [20]. Cortisol (R4866) and testosterone (R156/7) antibodies and corresponding horseradish peroxidase conjugates were obtained from C. Munro Clinical Endocrinology Laboratory (University of California Davis, USA). Steroid standards were obtained from Steraloids Inc. (Newport, RI, USA).

### 2.4. Statistical Analysis

The variability of the stress hormones was measured by their percent coefficient of variation (%CV). A repeated measures ANOVA (week x day) was used in order to examine changes in sC, sT, T/C ratio and sIgA. Compliance to daily saliva collection was 75% for the control week and 80% for the competition week. While the overall compliance was high, the missed days did affect which data were used in the analysis. To control for missing data, a listwise deletion was applied so that participants with missing daily values were deleted from the analysis when running the repeated measures model. Therefore, the final number of participants with complete data that was used in this analysis was 23. Data were analyzed separately for the swimmers with complete data (*n* = 23), as well as for those swimmers who reported no URTI symptoms during the control week (*n* = 18). Paired *t*-tests were used to compare the mean weekly responses of each marker. Pearson's product moment correlations were used to determine potential associations between markers of biological stress and mucosal immunity. An alpha level of <0.05 was used as the criterion for significance for all statistical analyses, which were conducted using SPSS version 19 for Windows (SPSS Inc., USA).

## 3. Results

Participants' age and physical characteristics are presented in Table 1. There was a significant difference in % BF between boys and girls. Sixty percent (60%) of the boys were classified as late pubertal (Tanner stage 4) while the girls were less mature as a group; 38% early pubertal (Tanner stage 2), 31% pubertal (Tanner stage 3), and 31% late pubertal. Despite these differences, when age, pubertal status, and sex were entered in the repeated measures model as potential covariates, no significant effect or interaction was found. In addition, although the boys had consistently 4–6% higher sT levels than the girls, this difference was not significant and there were no other significant differences in the daily hormonal levels between boys and girls. Therefore, the data were pooled together for the final analysis.

**Figure 1:**
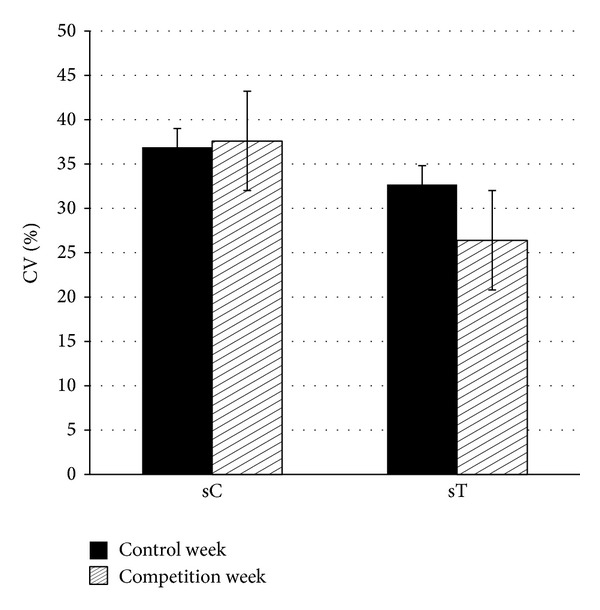
Percent coefficient of variability (%CV) of salivary cortisol (sC) and salivary testosterone (sT) during the control and competition week. Values are mean ± SE.

Weekly mean values of hormonal and immune biomarkers are presented in Table 2. Salivary testosterone (sT) was significantly lower during the experimental week in both boys and girls while the 13% increase of sIgA was not statistically significant. The percent coefficient of variation (%CV) of the hormones was not different between weeks (Figure 1) indicating no changes in their weekly variability.

**Figure 2:**
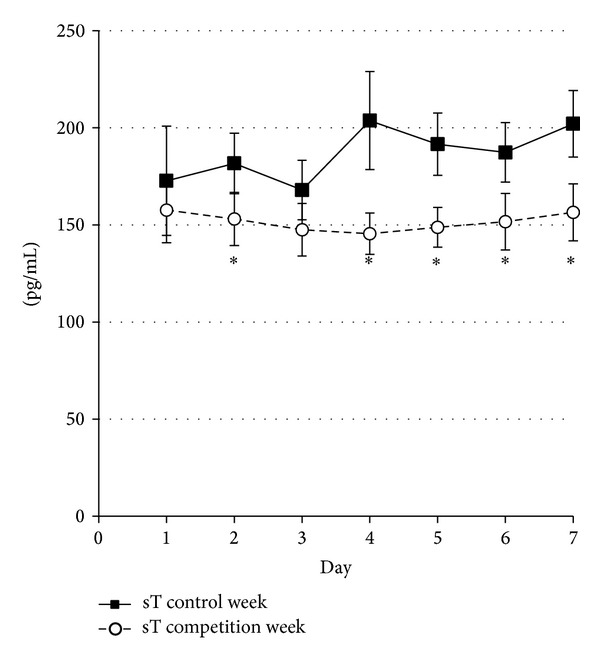
Salivary testosterone (sT) levels during the control and competition week. ^∗^Significant differences between control and experimental week (*P* < 0.05). Values are mean ± SE.

Repeated measures, two-way (week x day) ANOVA showed no significant effect of week or day for sC, T/C, and sIgA. Testosterone, on the other hand, showed a significant (*P* < 0.05) week effect with lower levels during the competition week for days 2, 4, 5, 6, and 7 (Figure 2).

The anxiety scores were within typical values with no difference between weeks. The overall anxiety score for the control week was 26.8 ± 1.89 while swimmers reported similar anxiety levels during the week of competition, 27.8 ± 1.8. In addition, all swimmers improved their performance times. Specifically, taking into account all events performed during the two days of competition, the mean percent improvement between seeding times and final times was 4.9 sec (SD 0.4) or 6.2% (SD 3.1).

In general, the correlation analysis indicated that during the control week, the number of days with URTI symptoms was positively correlated with the weekly mean sC (*r* = 0.45; *P* < 0.05) and sIgA (*r* = 0.46; *P* < 0.01) and negatively correlated with T/C ratio (*r* = 0.49; *P* < 0.05). During the competition week, number of days with URTI symptoms was positively correlated with sC (*r* = 0.50; *P* < 0.05). Moreover, URTI incidence was a significant covariate in the repeated measures ANOVA analysis although the prevalence of URTI was relatively low for both weeks; 5 of the 23 swimmers reported URTIs during the control week with a mean of 3.0 ± 1.0 days with URTI symptoms, and 5 other swimmers reported URTIs during the week of competition, with a mean of 3.8 ± 1.0 days with URTI symptoms. Therefore, we ran a separate analysis for those participants who reported no URTI symptoms at the beginning of the study (first week) (*n* = 18). A similar pattern of results was observed whether analysis was performed on the total cohort of swimmers or on the subsample with no URTI symptoms. This indicates that the results were independent of the initial immune state of the swimmers.

## 4. Discussion

This is the first study to examine biological stress and sIgA, a marker of mucosal immunity, in adolescent swimmers during a week leading to a competition as compared to a typical, control week. Contrary to our expectation, there was no difference in sC levels and no change in its variability between a typical, noncompetition week and a week leading to and including a significant competition in this group of young swimmers. Similarly, there were no significant differences in sIgA and incidence of URTI between weeks, and so no relationship was found between stress and immunity in these young swimmers. However, testosterone levels were lower during the competition week compared to the control week, along with a 7%, nonsignificant decrease in the weekly T/C. This may reflect some residual fatigue from previously intense training or insufficient/late tapering that might have affected the anabolic response of the swimmers while preparing for competition. This hormonal state, on the other hand, did not have a negative effect on the swimmers' performance.

### 4.1. Hormonal Response

On average, the observed cortisol levels corresponded to the typical range of sC values for this specific age (11–15 years old) with no differences between boys and girls [21, 22]. It was hypothesized that there would be an increased variability in stress hormones with sC gradually increasing and the T/C ratio decreasing the week of competition. However, the results showed that neither the absolute salivary cortisol levels nor the % CV of sC significantly differed between the control and the competition week. There are two possible explanations for the lack of difference in cortisol levels between the two weeks; either the control week did not reflect the swimmers' true baseline levels, or this major competition was insufficient to induce a significant stress response to the young athletes. This finding is in agreement with what was observed in adult athletes prior to an endurance running competition [23]. Nevertheless, our swimmers did not exhibit a significant cortisol change even during the days of competition, which is in contrast with the majority of the adult literature that has reported an increase of cortisol in the days of significant competitions in comparison to typical resting days [6, 7, 24, 25].

It is possible that the reduced training volume during the competition week masked the competition effect on salivary cortisol keeping it at the same level as when the training volume was higher during the control week. Given that training volume was lower by 25%, one would expect cortisol to decrease during the competition week as previously suggested [26]. In their study, Filaire et al. [26] found that cortisol decreased in response to recovery periods (tapering) in comparison to periods of intense training in soccer players of various ages. However, other studies investigating the cortisol responses of young athletes to training and/or competition have reported contradictory results [6, 27, 28]. Mortatti et al. [28] investigated the impact of a competition and training on hormonal and immune parameters in young soccer players over a period of seven games in 20 days and found no significant changes in sC levels, despite increments in training load. Similarly, when comparing sC levels before and after 16 weeks of training and competition in young female tennis players, Filaire et al. [29] found that there was a reduction in this parameter at awakening.

Salivary testosterone significantly decreased the week of competition in comparison to the control week, indicating that the majority of the swimmers had a compromised anabolic response. Testosterone's dynamics prior to a competition are not well established in the literature. Filaire et al. [6] found that salivary testosterone did not change before a competition in adult judoka. Others, however, have reported that testosterone responses in rugby players before and after a match were dependent on the importance of the match; the concentrations increased when the competition generated an important stress and decreased when the psychological conditions remained relatively stable [30]. According to Maso et al. [31], testosterone is influenced by tiredness with low testosterone levels being an indication of fatigue. Thus, the lower testosterone levels during the experimental week may reflect a residual effect of the intense training prior to the week of competition, and insufficient or late tapering the days prior to competition. In a recent study, Papacosta et al. [32] examined the time-course of change of sT, sC, and sIgA, mood state and performance during a 2-week taper in adult judoka, and found that changes in hormonal responses precede enhancements in performance and mucosal immunity, suggesting that judoka should taper for at least a week prior to competition. However, there are no studies in children and adolescent athletes on tapering and performance so it is unclear if one week of tapering was physiologically sufficient for our swimmers. On the other hand, all swimmers improved their times at the competition suggesting no direct, detrimental effect on performance.

In support to this point, T/C ratio decreased by 7% during the competition week. According to Filaire et al. [33], this ratio is considered to reflect the states of anabolism when it is high and the states of catabolism when it drops by 30% or more. Evidence of the same study shows that a decreased T/C ratio prior to competition does not necessarily lead to a decrease in the team's performance [33]. Given the slight decrease of the ratio the week of competition, it is not surprising that all swimmers improved their times.

### 4.2. Interaction between Markers of Biological Stress and Mucosal Immunity

The results did not support our hypothesis that sIgA counts would decrease as competition approached. In fact, during the competition week, sIgA slightly increased. Nevertheless, no immunosuppressive effect was observed in this population. Furthermore, although salivary cortisol was positively correlated with URTI, we found no relationship between stress and sIgA in this age group. Therefore, this correlation may indicate that the body is simply under stress when producing antibodies to fight a pathogen. Numerous studies have tried to examine this relationship although there seems to be no unanimity pertaining to this association. Cieslak et al. [34] suggested that stress does not have a negative effect on mucosal immunity in children, while physical activity seems to be positively associated with it. Tiollier et al. [35], who also found no significant correlation between salivary cortisol and sIgA, have suggested that cortisol levels do not differ between healthy and ill adults. However, studies in adult basketball or volleyball players have reported increased cortisol and suppressed immunity both during a competitive season [8] and before a significant match [7]. Despite these reports in adult athletes, the effect of stress on the immune system in younger athletes is less evident and requires further study.

## 5. Conclusions

Our findings indicate that mucosal immunity was unaffected in this group of young swimmers during a week leading to competition. The normal anxiety scores in conjunction with the unchanged cortisol levels and variability of both cortisol and T/C ratio during the study suggest that swimmers were not significantly stressed by the swim meet. Based on their training and competition background, it is possible that either these high performance swimmers were familiar with the conditions of a significant swim meet, or that the reduced amount of training (tapering) started late or was insufficient to induce a decrease in cortisol levels. Alternatively, the tapering effect on cortisol counteracted the anticipated competition-induced increase in cortisol and explains why cortisol variability also did not increase. In the week of competition, however, salivary testosterone decreased. The lower testosterone is attributed to the fact that swimmers had experienced a protracted intense training the weeks before the competition with insufficient or late tapering and, therefore, their testosterone levels decreased as a subsequent phenomenon of fatigue. However, this small shift in their apparent metabolic state did not negatively influence the swimmers' performance in the competition.

## Figures and Tables

**Table 1:** Subjects' physical characteristics (mean ± SE).

Variables	Boys *n* = 10	Girls *n* = 13	Total cohort *n* = 23
Age	13.9 ± 0.3	13.5 ± 0.4	13.6 ± 0.2
Body mass (kg)	56.4 ± 3.7	55.0 ± 3.0	55.5 ± 2.3
Body height (cm)	168.5 ± 3.8	164.3 ± 2.2	166.1 ± 2.1
Body fat (%)	11.4 ± 0.9	18.9 ± 1.3∗	15.6 ± 1.1

*P ≤ 0.05 difference between boys and girls.

**Table 2:** Salivary concentrations of hormonal and immune markers for the control and the competition week in adolescent swimmers (mean weekly values ± SE).

Variables	Control week	Competition week
	(*N* = 23)	(*N* = 23)
Cortisol (ng/mL)	2.7 ± 0.2	2.5 ± 0.2
Testosterone (pg/mL)	187.7 ± 13.5	154.5 ± 11.3∗
Testosterone/cortisol (ratio)	83.4 ± 7.0	77.9 ± 7.7
Immunoglobulin A (*μ*g/mL)	47.9 ± 4.4	54.9 ± 5.2

*P ≤ 0.05 difference between control and competition week.
